# 

**Published:** 1864-11

**Authors:** 


113 Ninth Street, New York, Oct. 14, 1864

Dr. J. Taft :—*Dear Sir*—By virtue of authority conferred upon us, as a committee created by the New York Society of Dental Surgeons, we send you for publication in the November No. of the Dental Register a list of subjects upon which Essays will be presented and read at the regular meetings of said Society.

Appended will also be found the names of gentlemen reading such papers and the date of their presentation :

Nov. 9th, 1864. Anatomy of the Mouth. Thomas Rowe.
Nov. 23d. Origin and Formature of Teeth. W. H. Atkinson.
Dec. 7th. Pathology of the Teeth. A. C. Castle.
Dec. 21st. Physiology of the Teeth. C. P. Fitch.
Jan. 4th, 1865. Periodontitus. A. L. Northrop.
Jan. 18th. 1st and 2d Dentition. W. C. Horne.
Feb. 1st. Artificial Dentures. J. M. Crowell, John Allen, and J. C. Robbins.

Feb. 15th. Duties of Private Dental Preceptors. C. A. MARVIN,

March 1st. Diseases of the Gums. J. S. LATIMER.

March 15th. Relations of Dentistry to General Medicine. W. H. ATKINSON, A. C. CASTLE, C. P. FITCH.

March 29. Hypertrophy. W. H. ATKINSON.

April 12th. Cleft Palate. N. W. KINGSLEY.

April 26th. Dental Etiquette. W. B. HURD.

May 10th. Diseases of the Antrum. J. S. LATIMER.

May 24th. Filling Teeth with the Mallet. G. A. MILLS.

June 7th. Irregularities of the Teeth; their Causes and Treatment. WM. H. ALLEN.

June 21st. Dental Instruments. FRANK ABBOTT.

July 5th. Anæsthesia and Anæsthetics. W. C. HORNE.

July 19th. Dental Hygiene. C. E. FRANCIS.

C. P. FITCH,<br>

W. H. ATKINSON, } *Committee.*<br>

J. M. CROWELL,

